# Unraveling the bioactivity of anticancer peptides as deduced from machine learning

**DOI:** 10.17179/excli2018-1447

**Published:** 2018-07-25

**Authors:** Watshara Shoombuatong, Nalini Schaduangrat, Chanin Nantasenamat

**Affiliations:** 1 Center of Data Mining and Biomedical Informatics, Faculty of Medical Technology, Mahidol University, Bangkok 10700, Thailand

**Keywords:** cancer, anticancer, antitumor, anticancer peptides, host defense peptides, bioactivity, machine learning, QSAR

## Abstract

Cancer imposes a global health burden as it represents one of the leading causes of morbidity and mortality while also giving rise to significant economic burden owing to the associated expenditures for its monitoring and treatment. In spite of advancements in cancer therapy, the low success rate and recurrence of tumor has necessitated the ongoing search for new therapeutic agents. Aside from drugs based on small molecules and protein-based biopharmaceuticals, there has been an intense effort geared towards the development of peptide-based therapeutics owing to its favorable and intrinsic properties of being relatively small, highly selective, potent, safe and low in production costs. In spite of these advantages, there are several inherent weaknesses that are in need of attention in the design and development of therapeutic peptides. An abundance of data on bioactive and therapeutic peptides have been accumulated over the years and the burgeoning area of artificial intelligence has set the stage for the lucrative utilization of machine learning to make sense of these large and high-dimensional data. This review summarizes the current state-of-the-art on the application of machine learning for studying the bioactivity of anticancer peptides along with future outlook of the field. Data and R codes used in the analysis herein are available on GitHub at https://github.com/Shoombuatong2527/anticancer-peptides-review.

## Introduction

Cancer is now regarded as the second leading cause of death, and remains a major cause of morbidity throughout the world (Arnold et al., 2015[4]) with lung, liver, colorectal, stomach and breast cancer representing the most common types of cancers occurring worldwide (WHO, 2018[101]). Estimates from GLOBOCAN indicate that about 14.1 million new cancer cases encompassed approximately 8.8 million deaths in 2015 (Ferlay et al., 2015[17]; WHO, 2018[101]). In addition, the main mechanisms by which cancers are formed include abnormal, uncontrollable cell growth that leads to the formation of tumors which can then undergo angiogenesis and continue to become metastatic (Felício et al., 2017[16]). Despite recent advances in cancer treatments, such as radiation therapy, targeted therapy or chemotherapeutic agents (Thundimadathil 2012[86]), they have a relatively low success rate and present a risk of recurrence. For instance, the process of killing cancer using chemotherapeutic agents is often associated with deleterious effects, including damages to normal cells and tissues, and lead to chemical resistance whereby adaptation mutations of cancer cells may occur (Hoskin and Ramamoorthy 2008[26]). Therefore, the discovery and development of a new class of anticancer drugs has become crucial. Furthermore, the situation has become worse due to the fact that many new cancers arise from bacterial and viral etiological agents (Vedham et al., 2014[93]). This fact coupled with the increase in antimicrobial resistance (AMR), especially the multidrug resistant variants, has raised concern. To this effect, the WHO has emphasized an urgency for the discovery of new therapeutic agents (WHO, 2018[100]).

**Figure 1:**
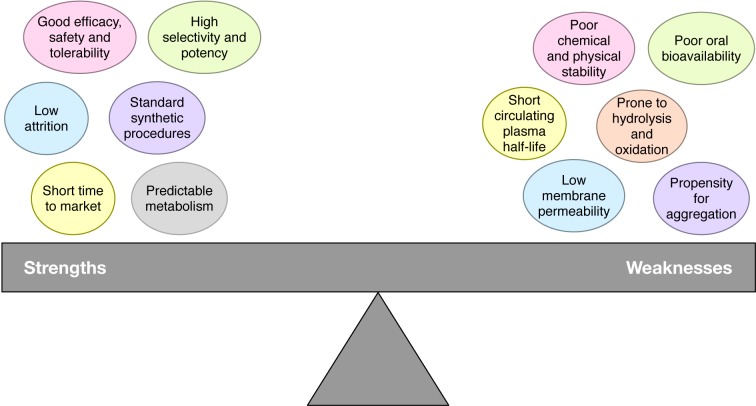
Strengths and weaknesses of therapeutic peptides. Concepts summarized from Fosgerau and Hoffmann, 2015.

Peptide therapeutics have attracted great interest for development as drug candidates as they are regarded to be safe, efficacious, highly selective with good tolerability as well as exhibit attractive pharmacological profiles (Craik et al., 2013[10]; Vlieghe et al., 2010[95]; Lau and Dunn, 2018[36]; Fosgerau and Hoffmann, 2015[19]). A summary on the strengths and weaknesses of therapeutic peptides are provided in Figure 1(Fig. 1) (Reference in Figure 1: Fosgerau and Hoffmann, 2015[19]). Owing to their intrinsically smaller size as compared to protein-based biopharmaceuticals, peptides are therefore more economical to produce due to lesser production complexity (Fosgerau and Hoffmann, 2015[19]) while at the same time possess more agility in their pharmacokinetics. The aforementioned properties are distinguishing features that set them apart from small molecules-based drugs and protein-based therapeutics. Thus far, there are more than 7,000 naturally occurring peptides in existence that have been shown to afford a wide range of bioactivities (e.g. tumor homing, antihypertensive, antiparasitic, antiviral, antiangiogenic, antibiofilm, antimicrobial, anticancer, etc.) that can consequently be applied to target various diseases such as cancer, diabetes, cardiovascular diseases, etc. (O'Brien-Simpson et al., 2018[62]; Jin and Weinberg 2018[30]; Karpiński and Adamczak 2018[32]). As of now, 60 peptide-based drugs have been FDA-approved (Usmani et al., 2017[92]) while another 150 peptides (Lau and Dunn 2018[36]) are currently in the pipeline of preclinical and clinical studies.

The breakthrough discovery of cecropin, the first antimicrobial peptide (AMP) (i.e. isolated from injecting silk moth, *Hyalophora cecropia*, with bacteria) was reported by Steiner et al. (1981[85]). In another landmark study conducted by Zasloff (1987[104]), AMPs from the African clawed frog, *Xenopus laevis*, were isolated and characterized for their role in the immune defense and is known as magainins. Since then, thousands of AMPs have been found in almost all living organisms such as plants, bacteria, fungi, animals etc. (Li et al., 2012[44]). Over the past decade, the use of AMPs as therapeutic agents for treating diseases have increased constantly.

The process of understanding the importance of AMPs might be useful for the discovery of new and resistance-free therapies for infectious as well as non-infectious diseases. Antimicrobial peptides constitute a mechanism of immune defense of the innate immune system with low antigenicity (Iwasaki et al., 2009[28]; Pasupuleti et al., 2012[66]) that can be found in numerous eukaryotic organisms of different species (Reddy et al., 2004[71]). More recently, research on AMPs have elucidated that these peptides also provide anticancer activity and thus termed anticancer peptides (ACPs). ACPs have been found to exhibit short time-frame of interaction (i.e. decreases the probability of resistance), low toxicity (i.e. not devoid of side effects as it may harm normal cells), specificity, good solubility as well as good tumor penetration thereby indicating the great potential for the use of ACPs in cancer therapy (Domalaon et al., 2016[13]; Gaspar et al., 2015[21]; Figueiredo et al., 2014[18]; Riedl et al., 2011[72]). 

Since they are not traditional drugs, the clinical development of therapeutic peptides face numerous challenges owing to their weaknesses as summarized in Figure 1(Fig. 1). Stability of peptides (i.e. lack of correlation between *in vitro* experiments and its efficacy in *in vivo* models) is a challenging issue. In spite of this, promising results have been sparingly been demonstrated in some animal studies (Makobongo et al., 2012[48]; Deslouches et al., 2007[12]; Berge et al., 2010[5]; Camilio et al., 2014[7]; Makovitzki et al., 2009[49]) in which good efficacy of peptides were able to establish *in vitro* stability with bioavailability in animal models. Another major drawback of therapeutic peptides is their poor oral bioavailability. This can be addressed by conjugating the peptide with a delivery system that allows it to bypass the digestive system and thus, enhance the pharmacokinetic properties of such peptides. Several studies have been conducted on the modifications and/or conjugations (e.g. substitution with non-canonical amino acid, peptide-peptide hybridization, target or polymer modification, PEGylation etc.) of therapeutic peptides (Narayana et al., 2015[59]; Braunstein et al., 2004[6]; Papo and Shai, 2003[65]; Hu et al., 2016[27]; Spinks et al., 2017[84]; Kelly et al., 2016[33]; Li et al., 2016[41]). Another concern is the short half-life of peptides. However, it should be noted that it is this particular characteristic of therapeutic peptides that allows it to escape resistance unlike other oncogenic therapies. However, research on improving the half-life of peptides without compromising their potency is currently an active area of research (Hao et al., 2015[25]; Podust et al., 2013;[68] Schellenberger et al., 2009[74]; Garay et al., 2012[20]; Penchala et al., 2015[67]). Despite some limitations, no other class of peptides have been able to surpass the multi-functionality of bioactive/therapeutic peptides and thus, these peptides possess high potential for use in many avenues of clinical applications.

The post-genomic era has brought about the birth of several omics (e.g. peptidomics, proteomics, glycomics, transcriptomics, interactomics, etc.) in our attempts to understand the fundamentals of life and how we can contribute to sustainability and the improvement of the quality of life (i.e. development of new diagnostics, therapeutics, etc.). These data are amassing at an exponential rate with no slowing down in hindsight, which sets the stage for the utilization of machine learning in making sense of these data and translating them into useful and actionable insights. There have been extensive reports on the utilization of machine learning approaches for correlating the sequences of therapeutic peptides with their biological activity (Shi et al., 1998[75]; Nagarajan et al., 2006[56]; Alam and Khan, 2014[2]; Mohseni Bababdani and Mousavi, 2013[55]; Tong et al., 2014[87]; Li et al., 2017[42]). A review of the literature indicated that there are currently no review articles concerning the use of machine learning and quantitative structure-activity relationship (QSAR) as applied to therapeutic peptides. However, there are a few review articles examining the use of QSAR for studying the biological activity of peptides at the general level particularly with emphasis on food protein-derived bioactive peptides (Nongonierma and FitzGerald 2016[61]), peptides in general (as well as proteins and nucleic acids) (Zhou et al., 2008[106]), peptides in general (as well as chemical molecules and proteins) (Du et al., 2008[14]). In a series of recent articles, Lee et al. (2016[37], 2017[38], 2018[39]) examined another facet on the use of machine learning (i.e. particularly support vector machine) together with targeted experiments (i.e. killing assays and small-angle X-ray scattering (SAXS) experiments) to explore the membrane activity in undiscovered peptide sequence space in which the aim was not on the antimicrobial activity but on the membrane curvature that is necessary for the activity and the subsequent relationship to sequence homology.

To the best of our knowledge, this review article represents the first systematic review on the utilization of machine learning for studying the bioactivity of anticancer peptides. It is hoped that this review would help contribute to further growth and expansion of the field by providing readers with the current state-of-the-art of the field as well as expected future trends and outlook.

## Anticancer Peptides

ACPs are small peptides that usually contain 5 to 50 amino acid residues while possessing high hydrophobicity and a positive net charge (i.e. cationic in nature) (Melo et al., 2011[53]). Thus, ACPs can interact with anionic cell membrane components of cancer cells and then selectively kill cancer cells. Additionally, ACPs can interfere with cancer cells by causing apoptosis mediated via mitochondrial disruption (Chen et al., 2001[9]), triggering necrosis via cell lysis (Papo et al., 2006[64]), stimulate the immune system of the host and prevent tumor angiogenesis (Al-Benna et al., 2011[3]). Being a subset of AMPs, the characteristics of ACPs are very similar. However, the physicochemical properties that drive some AMPs to possess anticancer activity is still unclear and more research is needed to understand these differences and help drive specific designs of ACPs. There have been a number of AMPs encountered in nature that possess anticancer activity, such as Aurein 1.2 (GLFDIIKKIAESF) a peptide isolated from a frog species (*Litoria aurea*), represents an AMP with antibacterial activity which was also highly active towards 55 different cancer cell lines *in vitro*, without any significant cytotoxicity (Rozek et al., 2000[73]; Dennison et al., 2007[11]; Giacometti et al., 2007[23]). In addition, the human neutrophil peptide-1 (HNP-1, ACYCRIPACIAGERRYGTCIYQGALWAFCC), represents an intrinsic AMP found in the innate immune system that plays a fundamental role in the defense against pathogens. The full mechanism of action of this peptide against cancer cells has not yet been established, but the activity has already been confirmed for different cancer cell lines, with very low cytotoxicity against healthy cells (McKeown et al., 2006[52]; Gaspar et al., 2015[21]). Furthermore, in terms of their structure, ACPs are mainly categorized as adopting either an α-helix (i.e cecropin, magainin, melittin, and buforin II) or β-sheet (i.e defensins (HNP-1, HNP-2 and HNP-3), lactoferricin B and tachyplesin) conformation due to their inability of fold into a well-defined structure in solution (Hoskin and Ramamoorthy, 2008[26]).

**Figure 2:**
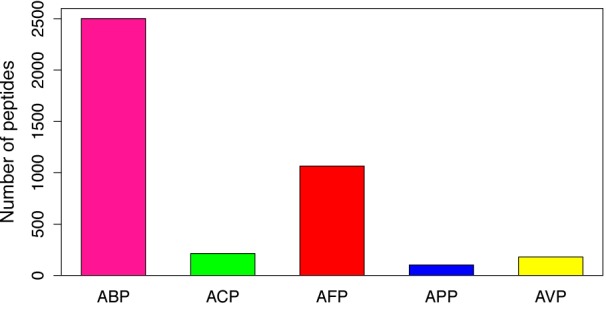
Bar plot of the number of antibacterial peptides (ABP), anticancer peptides (ACP), antifungal peptides (AFP), antiparasitic peptides (APP) and antiviral peptides (AVP). Data is collected from the antimicrobial peptide database (APD3) (Wang et al., 2016).

**Figure 3:**
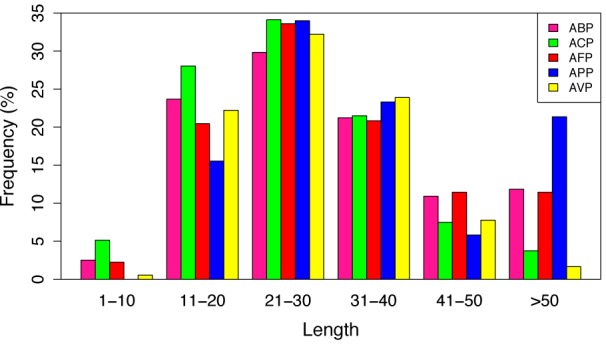
Bar plot showing the peptide length distribution in percentage for antibacterial peptides (ABP), anticancer peptides (ACP), antifungal peptides (AFP), antiparasitic peptides (APP) and antiviral peptides (AVP) collected from the Antimicrobial Peptide Database (APD3) (Wang et al., 2016).

**Figure 4:**
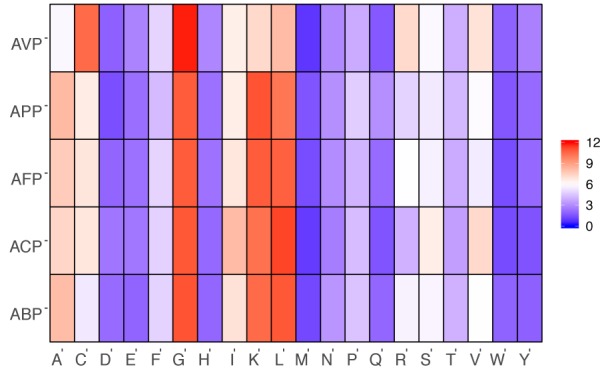
Heat map showing the amino acid compositions in percentage for antibacterial peptides (ABP), anticancer peptides (ACP), antifungal peptides (AFP), antiparasitic peptides (APP) and antiviral peptides (AVP). Data was collected from the Antimicrobial Peptide Database (APD3) (Wang et al., 2016).

**Figure 5:**
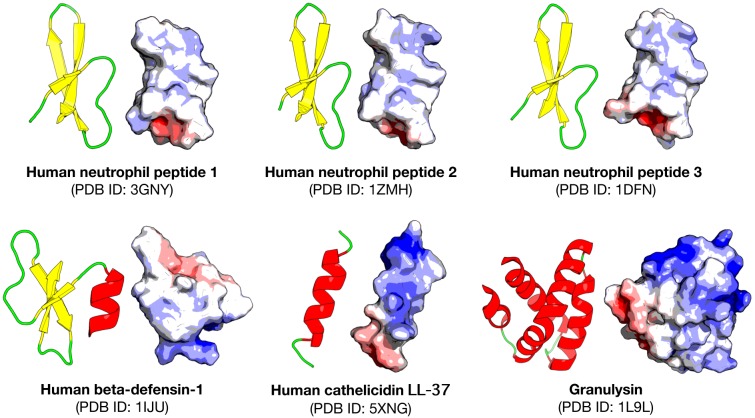
Structures of human-derived anticancer peptides

In the more recent years, a lot of focus has been placed on research into ACPs with the increase in AMP databases. One such database, the antimicrobial peptide database (APD3) (Wang et al., 2016[98]) (Available at http://aps.unmc.edu/AP/main.php) recorded as of May 10, 2018, a total of 2,981 AMPs, out of which, 215 have been classified as ACPs from various sources (animals, plants, bacteria, fungi and synthetic) (Figure 2(Fig. 2); Reference in Figure 2: Wang et al., 2016[98]). It should however, be noted that the different categories of the peptides (i.e. antibacterial, antiviral, antiparasitic, anticancer etc.) will contain peptides that overlap due to some exhibiting dual properties. In addition, upon further analysis of the peptide length determining anticancer activity, it was observed that (Figure 3(Fig. 3); Reference in Figure 3: Wang et al., 2016[98]), out of the 214 ACPs in the database (1 peptide “AP02769” contained a non-canonical amino acid and was excluded from the analysis) 73 (34.11 %) and 60 (28.04 %) were 21-30 and 11-20 amino acids in length, respectively. Furthermore, peptides of length between 21-30 amino acids exhibiting antibacterial, antifungal, antiparasitic and antiviral activities were observed at 746 (29.83 %), 358 (33.58 %), 35 (33.98 %) and 58 (32.22 %), respectively. Therefore, the most optimal peptide length for AMPs, especially for ACPs is 21-30 and hence, it is of great value to optimize the peptide length. Moreover, upon comparison of the most frequently observed amino acid residues constituting each category of AMPs (Figure 4(Fig. 4); Reference in Figure 4: Wang et al., 2016[98]), it can be seen that for ACP functioning, G (Gly at 10.88 %), K (Lys at 10.25 %) and L (Leu at 11.23 %) are the most predominant. Keeping with this tread, the most frequently observed amino acid for all categories of AMPs was Gly which was found at 10.98 %, 10.88 %, 10.79 %, 10.77 % and 11.82 % for ABPs, ACPs, AFPs, APPs and AVPs, respectively. Lysine was also abundantly found in most AMP categories, as indicated in Figure 4(Fig. 4), as it is a positively charged residue which could provide improvement in the cell and tissue penetrating properties of peptides (Li and Cho, 2012[45]). In addition, the hydrophobic residue Leucine was also predominant in all AMPs (10.88 %, 11.28 %, 10.66 %, 10.13 % and 8.11 % for ABPs, ACPs, AFPs, APPs and AVPs, respectively) which infers its importance in the structure and function of proteins (Jayaraj et al., 2009[29]) especially since therapeutic peptides usually contain around 50 % hydrophobic residues (Mansour et al., 2014[51]). Furthermore, the anticancer activity of human AMPs have only been evaluated for six peptide classes (Wang et al., 2016[98]) such as HNP-1, HNP-2, HNP-3, hBD-1, LL-37, and granulysin whose structures are shown in Figure 5(Fig. 5).

From thousands of available AMPs and many more that can be synthetically created, only a few have managed to reach clinical trials. Presently, only ten therapeutic peptides to treat various tumor types are currently being evaluated in various phases of preclinical and clinical trials (Felício et al., 2017[16]). This may be due to challenging developmental processes for turning these peptides into potent pharmaceutical drugs (e.g. cost of synthesis, peptide size, charge, and solubility) (Tørfoss et al., 2012[88]). However, with the increase in ACP research, more peptides may reach clinical trials in the future. With the help of synthetic approaches, peptide sequences could be altered so as to enhance their anticancer properties. But, the effect of these structural modifications on the physicochemical properties will need to be elucidated. Recently, these types of studies have increasingly made use of computational approaches (Prada-Gracia et al., 2016[69]; Maccari et al., 2015[47]; Kliger, 2010[35]; Tyagi et al., 2013[90]; Simeon et al., 2017[82]). In addition, several databases exist which have pooled the data of existing sequences that pertain to bioactive or therapeutic peptides. Some of the selected databases are described in Table 1(Tab. 1) (References in Table 1: Wang et al., 2016[98]; Minkiewicz et al., 2008[54]; Waghu et al., 2016[96]; Tyagi et al., 2015[91]; Fan et al., 2016[15]; Zhao et al., 2013[105]; Liu et al., 2008[46]; Singh et al., 2016[83]; Usmani et al., 2017[92]), where, out of all of the individual databases, only one named CancerPPD (Tyagi et al., 2015[91]) is available for ACPs. Five of the databases, are the most comprehensive databases for AMPs that have been combined from various organisms. In addition, it is noteworthy that only one database, THPdb (Usmani et al., 2017[92]) exists whereby data from FDA-approved peptides and proteins are available. Besides the databases mentioned in Table 1(Tab. 1), there is another database, TumorHoPe (Kapoor et al., 2012[31]), a database that provides information regarding experimentally characterized tumor targeting/homing peptides. These peptides recognize tumor tissues and tumor-associated micro-environments, including tumor metastasis. Thus, they can be used to deliver drugs selectively in tumors. In addition, a database catering to cell-penetrating peptides, CPPsite (Gautam et al., 2012[22]) that could also be advantageous for recognizing tumors as they exhibit similar properties such as short length (10-30 amino acids), are cationic or amphipathic (containing Arg and Lys residues), and high lipophilicity. More recently, a database dedicated to compiling structural information of bioactive peptides named StraPep (Wang et al., 2018[99]), which currently displays structures for 3,791 peptides as well as provides detailed information for each one (i.e. experimental structure, secondary structure, post-translational modification, etc.).

## Machine Learning

Machine learning is a natural outgrowth of the integration of computer science, mathematics and statistics that allows software application to become accurate in prediction without prior known information (Nasrabadi 2007[60]). The basic application of machine learning is to build algorithms that can formulate a data (a matrix *x_ij_*, where each row ***x***=(*x_i1_, x_i2_, x_i3_,…, x_ij_*) is a sample composed of *j* features) with its proper form and use a prediction model to elucidate an output.

The application of machine learning for correlating the relationship that exists between structures of biological and chemical entities (i.e. peptides and proteins for the former while small molecules for the latter) with their observed or experimentally measured biological activity gives rise to an exciting field of research known as quantitative structure-activity relationship (QSAR). The formulation of a QSAR model entails the generation of quantitative and/or qualitative description of the biological or chemical entities (i.e. known as descriptors) and their subsequent correlation with the biological activity (e.g. IC_50_, EC_50_, % activity, etc.) through the use of machine learning algorithms.

Details on the best practices for the development of QSAR models is beyond the scope of this review and readers are directed to previous literature (Nantasenamat and Prachayasittikul, 2015[57]; Tropsha, 2010[89]; Shoombuatong et al., 2017[80][81]). Briefly, characteristics of a robust QSAR model is best summarized by the OECD principles (OECD, 2014[63]) as outlined in Table 2(Tab. 2). In a nutshell, it can be clear that a robust QSAR model should be properly prepared and curated, afford good performance as well as being interpretable so as to facilitate the utilization of the model for gaining insights into the underlying biological activity.

## Model Set-up for Predicting Anticancer Peptides

Based on the prior knowledge of peptide sequence analysis, anticancer peptide prediction should be tackled in two associated ways: discriminating ACPs from non-ACPs and then predicting the anticancer activity of such ACPs. Due to the limitation of the experimental approach (e.g. slow and laborious process, expensive, difficulty in peptide purification etc.) for identifying the anticancer activity, computational tools for discriminating ACPs from non-ACPs is an essential way for saving the time-consuming and expensive cost. 

Typically, the computational tool construction based on machine learning algorithm consists of four main elements, e.g. data collection, feature representation, model construction and model evaluation (Shoombuatong et al., 2012[76], 2015[78][79], 2016[77], 2017[80][81]; Win et al., 2017[102]; Pratiwi et al., 2017[70]; Nantasenamat et al., 2015)[58]. In the point of view of machine learning, the use of reliable dataset plays a crucial role to obtain an efficient and generalized model. Previously, there have been many datasets that were used for developing various prediction models as shown in Table 3(Tab. 3) (References in Table 3: Tyagi et al., 2013[90]; Hajisharifi et al., 2014[24]; Vijayakumar and Ptv, 2015[94]; Chen et al., 2016[8]; Manavalan et al., 2017[50]). Meanwhile, the remaining important elements are listed in Tables 4(Tab. 4) and 5(Tab. 5). In the following section, a comprehensive summary of previous works in this field are highlighted.

## Machine Learning Models for the Prediction of Anticancer Peptide

Previously, a variety of computational approaches, including AntiCP (Tyagi et al., 2013[90]), Hajisharifi et al.,'s method (2014[24]), ACPP (Vijayakumar and Ptv, 2015[94]), iACP (Chen et al., 2016[8]), Feng et al.,'s method (Li and Wang, 2016[40]), iACP-GAEnsC (Akbar et al., 2017[1]), Fazlullah et al.,'s method (Khan et al., 2017[34]) and SAP (Xu et al., 2018[103]), have been developed, which will be discussed in the following section. Almost all of the existing methods were developed by using support vector machine (SVM) cooperating with various types of peptide features, except for iACP-GAEnsC (Akbar et al., 2017[1]) that was based on the ensemble approach. The overview of their datasets, type of features, machine learning algorithms and validation methods are shown in Table 5(Tab. 5) (References in Table 5: Tyagi et al., 2013[90]; Hajisharifi et al., 2014[24]; Vijayakumar and Ptv, 2015[94]; Chen et al., 2016[8]; Li and Wang, 2016[40]; Khan et al., 2017[34]; Akbar et al., 2017[1]; Manavalan et al., 2017[50]; Xu et al., 2018[103]). Meanwhile, Table 6(Tab. 6) (References in Table 6: Tyagi et al., 2013[90]; Hajisharifi et al., 2014[24]; Vijayakumar and Ptv 2015[94]; Chen et al., 2016[8]; Li and Wang 2016[40]; Khan et al., 2017[34]; Akbar et al., 2017[1]; Manavalan et al., 2017[50]; Xu et al., 2018[103]) lists the performance comparison among the existing methods as evaluated by 5-fold CV, 10-fold CV and jackknife test.

Tyagi et al. (2013[90]) first addressed this problem by using SVM-based predictor named AntiCP, in which TY1, TY2 and TY3 datasets were implemented with AAC, DPC and binary profile. The research however, does not specifically state the type of kernel function used. SVM model with ACC/DPC yielded prediction accuracies of 85.52 %/ 85.29 % and 75.70 %/75.20 % respectively, evaluated by a 10-fold CV method on TY1 and TY2 datasets. These results revealed that the importance of ACC feature for enhancing ACP prediction was not quite different from DPC feature. But, when binary (NT10) based models were applied, where NT10 was the first 10 residues and each amino acid was represented by (20*10)-dimensional vector, the accuracy improved to 91.44 %. Finally, SVM based on the NT10 models performed well with 89 % accuracy and 0.78 MCC on TY_IND dataset. Finally, a web-server (Available at http://crdd.osdd.net/raghava/anticp/) was developed to help experimental scientists in predicting minimum mutations required for improving anticancer potency, virtual screening of peptides for discovering novel anticancer peptides and scanning natural proteins for identification of ACPs.

Hajisharifi et al., (2014[24]) took advantage of PseAAC feature and local alignment kernels for improving the prediction performance of the model. In the study, the benchmark ZOH dataset was firstly created by collecting data from the antimicrobial peptide database (APD2) (Wang et al., 2009[97], available at http://aps.unmc.edu/AP/). The ZOH dataset consisted of 192 ACPs and 215 non-ACPs and then, to prevent an overestimation of prediction results due to highly similar sequences, peptides with more than 90 % similarity were removed from the initial ZOH dataset using CD-HIT (Li and Godzik, 2006[43]). Finally, a total of 138 ACPs and 206 non-ACPs were gained as summarized in Table 2(Tab. 2). SVM model conjunction with PseACC feature showed the values of accuracy, sensitivity, specificity and MCC of 83.82 %, 81.84 %, 85.36 % and 0.66, respectively, evaluated by a 5-fold CV procedure. Meanwhile, using a local alignment kernel yielded better prediction results than PseACC feature with improvements of > 6 % and 10 % on both Ac and MCC, respectively.

Only one year later, Vijayakumar and Ptv (2015[94]) utilized two powerful SVM and AdaBoost models cooperating with the protein relatedness measure (PRM) parameters called ACPP. The PRM feature represents each peptide with the degree distribution of amino acids deviating from a theoretical protein/peptide. To build a prediction model, SVM model with radial basis function (RBF) kernel and the tuning *cost* and *gamma* parameters of 2 and 0.0078, respectively, were used, while AdaBoost model based on the linear combination of simple weak classifiers with the tuning number of 10 iterations was applied. In this study, ACPP was evaluated with a 10-fold cross-validation method and independent test. SVM and AdaBoost were first carried out on the imbalanced dataset containing 217 ACPs and 3,979 non-ACPs as summarized in Table 2(Tab. 2). The prediction results showed that SVM and AdaBoost yielded MCC values as low as 0.59 and 0.57, while, the balanced dataset (217 peptide sequences on both ACPs and non-ACPs), yielded increased accuracies for SVM and AdaBoost of 0.92 and 0.88, respectively. Based on these results, the authors stated that the PRM feature adopted to classify ACPs from non-ACPs was effective. Although, in this study, a web-server was established at http://acpp.bicpu.edu.in/predict.php, however, it is currently unavailable.

In 2016, there were two different research groups that made efforts to develop ACP predictors, i.e. iACP (Chen et al., 2016[8]) and Feng et al.,'s method (Li and Wang, 2016[40]). Chen et al. (2016[8]) proposed an approach to take advantage of SVM model in conjunction with g-gap dipeptide compositions (g-gap DPC), where g = 0, 1, 2, 3 or 4 and g =0 is DPC, as well as working together with ANOVA (analysis of variance). Herein, SVM model with radial basis function (RBF) kernel and their optimal parameter of *cost* = 2 and *gamma* = 0.125 were used. The ANOVA approach via the incremental feature selection (IFS) was used for selecting informative features among g-gap DPCs. The process of determining the optimal number of features was conducted according to the following steps: (1) the feature with the highest F-score was selected as the input of SVM and the prediction performance assessed with 5-fold CV was calculated to evaluate the performance of this feature; (2) the feature with the second highest F-score was then combined with the first feature to form a new feature subset and the prediction performance with the criteria was still used to estimate the performance of the new feature subset; (3) this process was done when the prediction performance of 400 features were calculated. The highest accuracy of 94.77 % can be achieved by using g=1 and the 126 top-ranked informative features. Li and Wang (2016[40]) attempted to improve the prediction performance by using SVM model with hybrid composition, i.e. AAC, auto covariance of the average chemical shift (acACS) and reduced amino acid composition (RAAC). The parameters of RBF kernel used were tuned using the grid search method. Initial prediction results for their model using AAC on the ZOH dataset showed the value of accuracy and MCC of 91.86 % and 0.83, respectively. The second and third highest accuracies were obtained from using RACC (84.01 %) and asACS (82.56 %), respectively. Meanwhile, the combination features of AAC, RAAC and acACS performed best with 93.61 % accuracy and 0.87 MCC. The authors of this paper suggested that these combination features were helpful to the prediction of ACPs.

In 2017, three different ACP predictors were developed with various types of machine learning algorithms and peptide features, i.e. Khan et al.,'s method (2017[34]), iACP-GAEnsC (Akbar et al., 2017[1]) and MLACP (Manavalan et al., 2017[50]). Khan et al. (2017[34]) utilized SVM and *k*-nearest Neighbor (*k-*NN) models with a variety of peptide features, i.e. split amino acid composition (SAAC), DPC and PseAAC, to find the suitable feature for discriminating ACPs from non-ACPs. The total number of feature spaces of SAAC, DPC and PseAAC were 400, 62 and 60, respectively. To build prediction models, authors used RBF kernel to create SVM model, while euclidian distance was used to compute the distance among the peptide sequences. The optimum parameters of these two models were obtained during the training phase. The performance comparison evaluated by jackknife test demonstrated that SVM and *k-*NN models using SAAC outperformed the other two features with an accuracy of 93.31 % and 90.17 %. Akbar et al. (2017[1]) examined the ability of a variety of machine learning algorithms, i.e. SVM, random forest (RF), *k*-nearest Neighbor (*k-*NN), generalized neural network (GRNN), and probabilistic neural network (PNN). In this study, each peptide was represented by three different feature extraction schemes using RAAP, Pse-g-Gap dipeptide composition (Pse-g-gap DPC) and amphiphilic PseAAC (Am-PseACC). Finally, the evolution genetic algorithm was used to measure the diversity and optimum outcome or prediction results of the different methods called iACP-GAEnsC. Initial prediction results showed that using Am-PseACC with jackknife test achieved accuracies of 93.60 %, 90.41 %, 91.28 %, 86.33 % and 93.89 % for SVM, *k*-NN, PNN, RF, GRNN and GAEnsC, respectively. Their best accuracy of 94.45 % was achieved by using an ensemble approach with the merging of SVM, *k*-NN, PNN, RF and GRNN associated with a hybrid feature of RAAP, Pse-g-gap DPC and Am-PseACC. Manavalan et al. (2017[50]) developed machine learning-based methods (SVM and RF), named SVMACP, RFACP and MLACP using a combination of features, including ACC, DPC, PCP and ATC. The number of dimensions for ACC, DPC, PCP and ATC features were 20, 400, 11, 5, respectively. For each model, authors optimized the RF (*ntree* and* mtry*) and SVM (*cost* and *gamma*) parameters by using 10-fold CV on the TY3 dataset. In the case of using a single feature, RFACP and SVMACP yielded accuracies ranging from 81.4 %-86.8 % and 75.9 %-85.8 %, respectively. The best accuracy and MCC of 87.2 and 0.70, respectively, was achieved by using RF model with the combination feature of ACC, DPC, PCP and ATC.

Recently, Xu et al. (2018[103]) developed the MRMD method to select important features from g-gap DPC. The selected, informative feature was used as an input feature to train thethe SVM model called SAP. The paper does not specifically state the type of kernel function used. For a 5-fold CV, SAP using all 400 features yielded 91.86 % accuracy and 0.83 MCC, while using selected features offered a 90.70 % accuracy and 0.81 MCC. Furthermore, SAP was also compared with RF and LibD3D, where LibD3D is a selective ensemble model. The overall accuracy comparison showed that SAP (91.78 %) was quite comparable with RF (91.88 %) and LibD3D (89.24 %) models. 

The aforementioned articles showed promising results in the use of various types of machine learning algorithms and peptides features as summarized in Tables 5(Tab. 5) and 6(Tab. 6). As seen in Table 3(Tab. 3), the ZOH is known as the valid benchmark dataset used for developing various prediction models (Hajisharifi et al., 2014[24]; Chen et al., 2016[8]; Xu et al., 2018[103]; Khan et al., 2017[34]; Akbar et al., 2017[1]). Amongst these methods, iACP (Chen et al., 2016[8]) and iACP-GAEnsC (Akbar et al., 2017[1]) showed their best predictive accuracies of 94.77 % and 96.45 % as evaluated by 5-fold CV and jackknife test procedures, respectively. In addition, iACP revealed its efficiency by carrying out an independent WC_IND data achieving an accuracy and MCC of 92.67 % and 0.85, respectively. Considering that the independent test is the most rigorous cross-validation method, it might be stated that iACP (Chen et al., 2016[8]) was superior to other prediction methods as demonstrated in Table 5(Tab. 5). Amongst the existing methods, some of them (Tyagi et al., 2013[90]; Chen et al., 2016[8]; Manavalan et al., 2017[50]) determined the important amino acids and dipeptide that were enriched in anticancer peptides using componential analysis (Manavalan et al., 2017[50]; Tyagi et al., 2013[90]) and F-score (Chen et al., 2016[8]).

## Biological Insights from Predictive Models

Feature importance analysis from existing models (Tyagi et al., 2013[90]; Chen et al., 2016[8]; Manavalan et al., 2017[50] indicated that in general anticancer peptides are abundant in Cys, Glu, Phe, Gly, lle, Lys and Phe when compared to non-anticancer peptides (Chen et al., 2016[8]). Particularly, Tyagi et al. (2013[90]) reported that Gly, Leu, Ala and Phe were preferential residues at the N-terminus of anticancer peptides while Val, Cys, Leu and Lys were likely to be found at the C-terminus. Furthermore, Manavalan et al. (2017[50]) revealed that the 10 top-ranking features in anticancer peptides were comprised of dipeptides rich in positively charged and aromatic residues (e.g. KK, AK, KL, AL, KA, KW, LA, LK, FA and LF). Moreover, it should also be noted that desirable trait for anticancer peptides is their cell penetrating ability such that they can specifically neutralize their target while maintaining low toxicity.

## Limitations of Current Machine Learning Models

The use of machine learning algorithm is one of the important factors in the steady growth of the field of anticancer drug discovery and development. Most of the reported anticancer peptide prediction methods were mainly developed in order to enhance the prediction accuracy by taking advantage of the complexity of prediction methods and the number of feature types. Overall, most research articles showed encouraging results with having satisfied accuracies of more than 90 %. Nevertheless, there is still room for development to improve the existing methods as useful and interpretable models for facilitating experimental scientists and related researchers as demonstrated by a series of recent publications (Shoombuatong et al., 2012[76], 2015[78][79], 2016[77], 2017[80][81]; Win et al., 2017[102]; Pratiwi et al., 2017[70]; Nantasenamat et al., 2015[58]) and summarized in comprehensive reviews (Nantasenamat et al., 2015[58]; Shoombuatong et al., 2017[80][81]).

In addition, the most commonly used benchmark dataset ZOH, (Hajisharifi et al., 2014[24]) consisted of 138 ACPs and 206 non-ACPs in which only ACPs were derived from the experimental verification method. It could be stated that existing methods developed by ZOH dataset might not be completely suited to accurately filter experimentally verified non-ACPs from ACPs. Furthermore, peptide features were intrinsically heterogeneous, noisy and multi-dimensional, but only a few existing methods (Chen et al., 2016[8]; Xu et al., 2018[103]) took advantage of feature selection techniques to qualify and rank the importance and the contributions of the features for the model performance. Thus, these method has utilized only partial information of the biological activity of ACP. It could be stated that the role of different types of peptide features contributing to the biological activity of anticancer peptide are still poorly understood. Additionally, a variety of methods were used to evaluate the prediction performance of ACP predictions as listed in Table 5(Tab. 5), including N-fold cross-validation, where N is 5 or 10, jackknife test and independent test. The independent test is an effective way to test the performance of a model in real-world applications and verify the generalization of a model, but only few existing methods (Tyagi et al., 2013[90]; Vijayakumar and Ptv, 2015[94]; Chen et al., 2016[8]; Li and Wang, 2016[40]) were assessed with this method. Finally, according to the fifth principle of OEC which states that, it is necessary and significant of an interpretable QSAR model to provide important factors that can enhance the biological activity of peptides or compounds. Amongst the existing methods, some of them (Tyagi et al., 2013[90]; Chen et al., 2016[8]; Manavalan et al., 2017[50]) provided the results of feature importance analysis by using componential analysis (Manavalan et al., 2017[50]; Tyagi et al., 2013[90]) and F-score (Chen et al., 2016[8]). However, they did not clearly mention which features contributed most to prediction performance. Moreover, the SVM model was not straight-forward enough to interpret the underlying biological implications of anticancer peptides.

## Conclusion

The success story of therapeutic peptides is starting to gain moment with more than 60 approved by the FDA and more than 150 peptides have reached pre-clinical and clinical stages. In addition, a literature review have indicated that there is a large volume of on-going studies being carried out in the field. In spite of the large sum of papers on the utilization of machine learning approaches for the development of QSAR models of bioactive and therapeutic peptides, however there are few review articles that examine the field in a systematic manner. It is the intent of this review article to fill this gap by providing readers with the current advancements pertaining to the current state-of-the-art on the prediction of anticancer peptides via the use of machine learning approaches.

A survey of existing QSAR models against anticancer peptides suggested that almost all provided reasonably high prediction accuracies and in spite of this, there are limiting factors that may hinder their full potential for application as follows:

1. Absence of experimentally verified non-anticancer peptides.
2. Inclusion of trivial and non-informative features during the model building process.
3. Lack of comprehensive evaluation method and failure to make use of interpretable learning methods.

In efforts to augment the robustness of the predictive model, herein are recommendations:

1. Increase the size of the peptide dataset by combining all data sources together as to capture as much as possible of the pattern of dataset for alleviating uncertainties in the prediction system.
2. Familiarize oneself with the background and details of the descriptors being used such that the resulting models could be interpreted in a meaningful manner as to gain biological insights for guiding further experiments.
3. Use interpretable learning algorithms as to allow the interpretation of important features responsible for the biological activity.
4. Ensure that the model is externally validated on an independent test set as well as defining the applicability domain of the model as to verify the ability of the model for extrapolation to future unknown data.
5. Ensure the reproducibility of constructed models such that interested users could extend the model further.
6. If possible, constructed models should be made publicly available in the form of webservers so as to facilitate easy access to the model's prediction capability.

## Conflict of interests

The authors declare that no competing interests exist.

## Acknowledgements

This work is supported by the Office of Higher Education Commission and the Thailand Research Fund (No. MRG6180226); the New Researcher Grant (A31/2561) from Mahidol University; and the Center of Excellence on Medical Biotechnology (CEMB), S&T Postgraduate Education and Research Development Office (PERDO), Office of Higher Education Commission (OHEC), Thailand. Partial support from the annual budget grant (B.E. 2557-2559) of Mahidol University is also acknowledged.

## Figures and Tables

**Table 1:**
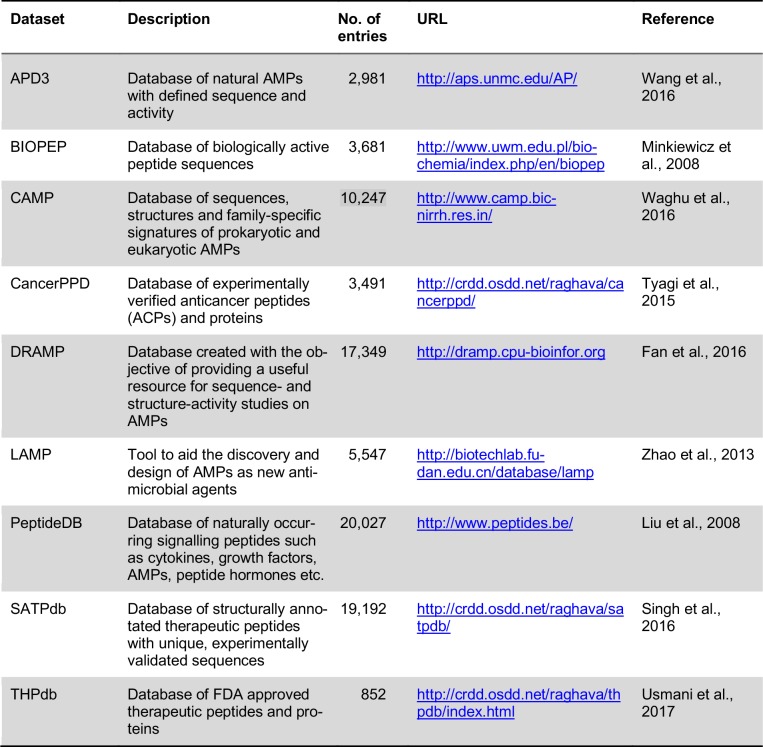
List of selected major databases available for bioactive and therapeutic peptides.

**Table 2:**
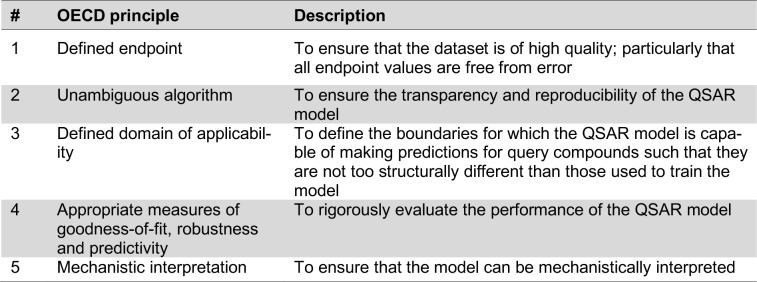
Summary of the OECD principles for the development of robust QSAR models.

**Table 3:**
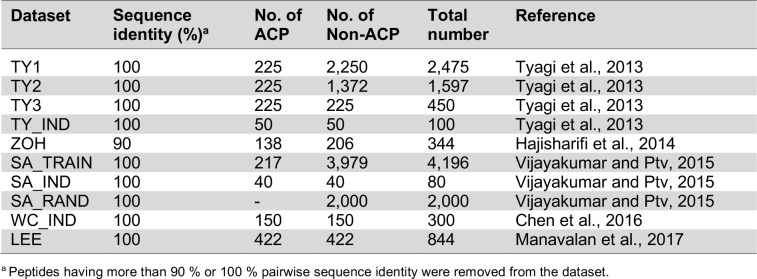
Summary of all datasets used in this research for evaluating anticancer peptide prediction.

**Table 4:**
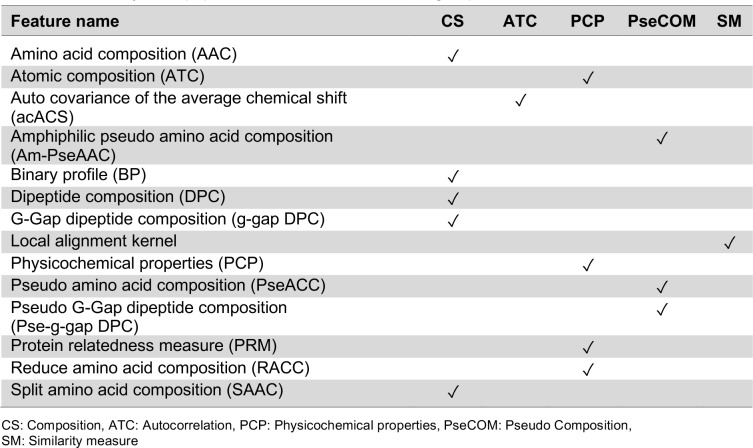
Summary of all peptide features and their feature groups in this research.

**Table 5:**
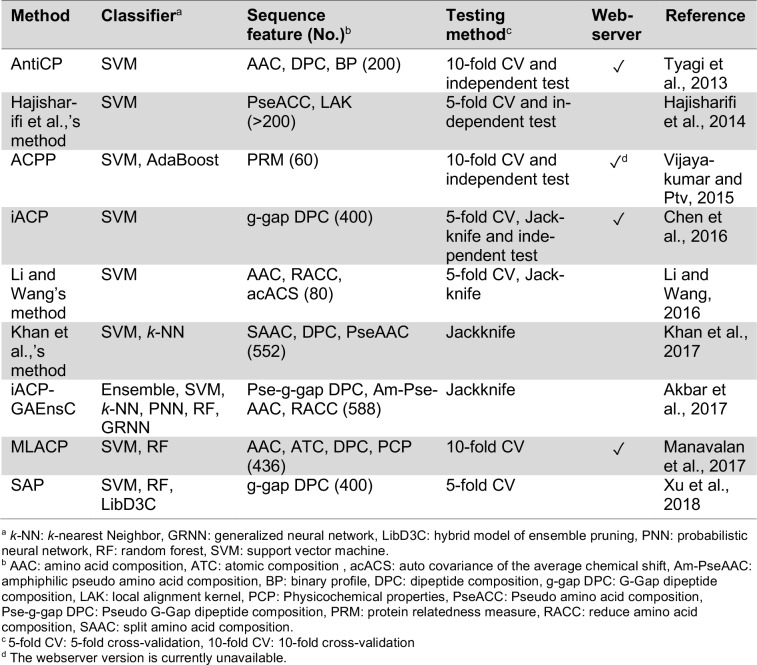
Summary of existing methods for predicting anticancer peptides.

**Table 6:**
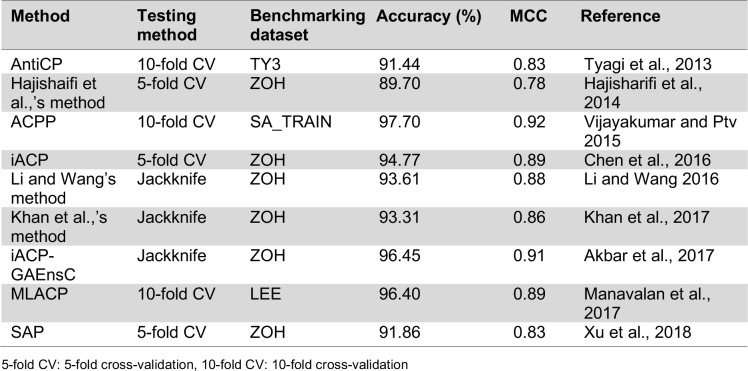
Performance benchmark comparing various computational methods evaluated by 5- and 10-fold cross-validation and jackknife test.
